# Comparative physical activity as a global question to assess physical activity among university students

**DOI:** 10.1186/s13102-021-00247-7

**Published:** 2021-03-02

**Authors:** Katharina Diehl, Alessia Brassat, Jennifer Hilger-Kolb

**Affiliations:** Mannheim Institute of Public Health, Social and Preventive Medicine, Medical Faculty Mannheim, Heidelberg University, Ludolf-Krehl-Straße 7-11, 68167 Mannheim, Germany

**Keywords:** Physical activity, Comparative optimism, Students, University

## Abstract

**Background:**

To assess physical activity (PA), a comparative measurement – evaluating one’s own PA compared to others – may be an appropriate method. In previous studies, the use of comparative measurements led to an effect known as unrealistic comparative optimism (UCO) – people being unrealistically optimistic about their behavior. Our aim was to use this comparative measurement in university students to quantify the prevalence of UCO at the group level and to draw conclusions on its validity.

**Methods:**

We used data from the Nutrition and Physical Activity in Adolescence Study (NuPhA), a cross-sectional online survey that included only self-reports (*n* = 689). To assess PA among students, they were asked to rate their PA level compared to that of their same-aged fellow students. In addition, we used the Godin-Shephard leisure-time PA questionnaire and other questions on PA for comparisons. We used bivariate and cluster-based analyses to identify potential UCO.

**Results:**

We found that UCO at the group level led to an uneven distribution, with a higher proportion of students who rated themselves as being more physically active than average. However, the individual assessment of PA with a single and simple comparative question seemed to be valid.

**Discussion:**

A global single comparative question seems useful for studies where PA is measured as a covariate in university students.

**Supplementary Information:**

The online version contains supplementary material available at 10.1186/s13102-021-00247-7.

## Background

Physical activity (PA) is a complex and multi-dimensional behavior [1] that includes different aspects such as exercise and household chores [2]. A widely used definition of PA was published by Caspersen et al. [3], who categorized PA in daily life into occupational, sports, conditioning, household, and other activities. This definition allows a clearer distinction between exercise and physical fitness.

Being a complex construct, PA is difficult to measure. Over the last few years, research has mostly concentrated on direct measurement methods such as questionnaires, extensive item batteries, and accelerometers. However, objective measures such as accelerometry and pedometry are cost- and time-consuming. Furthermore, their integration into studies that use only questionnaires with self-reports is difficult.

For studies whose focus is not to assess PA and those in which PA is only used as a covariate, reliable and validated PA assessment tools are needed. Therefore, different single item measures have been developed [4]. Sternfeld et al. [2] suggested that a comparative measurement of PA – defined as evaluating one’s own PA compared to other individuals – may be an appropriate and simple method to assess PA as a covariate in large epidemiologic studies. Sternfeld et al. [2] used a large and multiethnic sample of midlife women to assess comparative PA. However, they found that more women reported to be more physically active compared to same-aged women than to be less physically active [2]. The reason for this unexpected finding was that women who believe themselves to be more active were not balanced by those who reported a lower PA than average, thus, the average woman did not show an average PA. This effect is widely known as unrealistic comparative optimism (UCO) at the group level [5].

UCO describes that people tend to be unrealistically optimistic in their judgments about their own behavior and future life events compared to other individuals [6–8]. Shepperd et al. [9] describe it as the “erroneous estimate that one’s personal outcomes will be more favorable than the outcomes of peers” ([9], p.233). Individuals often underestimate their own possibility of experiencing negative events, such as cancer or car accidents and overestimate the possibility of positive events [9]. Therefore, individuals may overestimate their PA at the group level, that is, more individuals indicate being more physically active than being less physically active, compared to average.

These findings suggest that using a comparative measure might be problematic. Therefore, our aim was to make a first step towards validating a comparative PA measurement in specific target groups, in line with Sternfeld et al. [2]. We used this comparative measure in a Germany-wide quantitative online survey among university students from > 40 universities. To test concurrent validity, we analyzed associations between the comparative measurement and other self-reported PA variables (e.g., PA during the last week). In addition, by assessing this comparative measurement, we intended to quantify the prevalence of UCO at the group level. The existence of UCO at the group level has tremendous consequences for future prevention and health promotion measures, especially among university students.

## Methods

Our analyses are based on data from the cross-sectional NuPhA-Study (Nutrition and Physical Activity in Adolescence), an online survey among students at German universities. Data were collected from October 2014 to January 2015. In total, 689 students (aged 16–29 years, 69.5% female) from > 40 German universities completed the survey. Students were recruited via fliers, mailing lists, social networks, and announcements during lectures and seminars. Positive ethical approval was obtained from the Medical Ethics Committee of the Medical Faculty Mannheim, Heidelberg University (2013-634 N-MA).

To assess PA among students, they were asked to rate their *level of PA* compared to their same-aged fellow students by selecting one of the following response categories: much less, less, same as, more, or much more [2].

Furthermore, students answered questions on *paying attention to sufficient PA* (very much/much/neutral/less/much less), *physical performance* (very good/good/moderate/not really good/ not good at all), *PA during the last week* and *a normal week* (for at least 60 min on: 0 days/1 day/2 days/3 days/4 days/5 days/6 days/7 days), *regularity of sports activity with sweating or fast heartbeat* (often/sometimes/never), and *the amount of sports activity per week* (none/up to 1 h/1–2 h/2–4 h/more than 4 h). The questions on paying attention to sufficient PA and amount of sport activity per week were derived from the German Health Interview and Examination Survey for Adults (DEGS1) [10]. The question on physical performance was previously used in the German Health Interview and Examination Survey for Children and Adolescents (KiGGS) [11]. Both are nationwide representative surveys and essential component of the German health reporting system. PA during the last and a normal week is a questionnaire called the PACE+ Adolescent Physical Activity Measure [12], which is a valid and reliable measure in adolescents. The regularity of sport activity with sweating and fast heartbeat is an additional question of the Goding leisure-time exercise questionnaire [13], an instrument tested for reliability and validity in children, adolescents, and adults [14].

To assess self-reported leisure-time PA, we included the Godin-Shephard leisure-time PA questionnaire [15]. This questionnaire was used in other studies that focused on university students from different countries (e.g. [16, 17],) and has been shown to be a valid measure to group healthy adults into active and insufficiently active categories [18]. Test-retest reliability was good in previous studies [19] and the questionnaire has been validated with objective and other self-reported measures [19, 20].

The Godin-Shephard leisure-time PA questionnaire assesses self-reported strenuous, moderate, and mild exercise [15]. Participants indicate the times per week they spend each of these three forms of PA for more than 15 min. Based on these reports, a leisure-time activity score can be calculated using the formula: (strenuous PA × 9) + (moderate PA × 5) + (mild PA × 3) [15]. In addition, a health contribution score can be calculated by combining strenuous PA (× 9) with moderate PA (× 5) [15]. This metric value can be categorized into active (24 or more units), moderately active (14 to 23 units), and insufficiently active (less than 14 units) individuals [15]. For our analysis, we used *strenuous PA* (metric), *moderate PA* (metric), the *overall score* (metric), and the *health contribution score* (metric as well as categorial to underline dose-response between volume of PA and health benefits based on the Godin-Shephard leisure-time PA questionnaire [15]. In addition, *self-rated general, physical, and mental health* (very good/good vs. fair/poor/very poor) were measured.

For to describe our study sample, we report sex (female vs. male), age groups (≤ 20 vs. 21–22 vs. 23–24 vs. ≥ 25), family status (relationship vs. no relationship), immigrant background (yes vs. no), BMI calculated based on self-reported weight and height (underweight vs. normal weight vs. overweight), kind of university (university vs. university of applied sciences vs. dual university vs. others), field of study (Politics and Social Sciences vs. Education vs. Medicine and Health Sciences vs. Natural Sciences and Maths vs. Law vs. Linguistic and Cultural Studies vs. Psychology vs. Sport Sciences vs. Others), and current semester of the academic career (1–3 vs. 4–5 vs. 6–9 vs. more than 10).

To analyze sex differences in the comparative measurement, we calculated chi^2^-tests. Afterwards, we investigated associations between the comparative measurement and the above-mentioned variables on PA using chi^2^-statistics. For metric variables, we calculated the mean and conducted Kruskal-Wallis-H-Tests due to non-normal variable distributions.

In addition, we conducted a hierarchical cluster analysis to identify subgroups of students based on the variables of paying attention to sufficient PA, physical performance, PA during the last week and a normal week, the regularity of sports activity with sweating or fast heartbeat, and the amount of sports activity per week in order to group students according to their answers to the comparative question [21]. We used the Ward method (distance measure: Euclidean distance) and calculated standardized z-scores, due to the different scales. The analysis revealed a three cluster solution. For all tests in this study, the pre-defined level of statistical significance was *p* < 0.05. We used SPSS Version 24 (IBM for statistical analyses).

## Results

The majority of our sample was female (69.5%; Table 1), with a mean age of 22.7 years. Altogether, 13.9% had an immigrant background, and the majority studied medicine and health sciences.

**Table 1:** Description of the study sample

	Total [% (n)]	Male [% (n)]	Female [% (n)]	*p*-value
Sex	100.0 (689)	30.5 (210)	69.5 (479)	
Age				.081
≤ 20	24.2 (167)	19.0 (40)	26.5 (127)	
21–22	24.7 (170)	22.9 (48)	25.5 (122)	
23–24	27.3 (188)	30.5 (64)	25.9 (124)	
≥ 25	23.8 (164)	27.6 (58)	22.1 (106)	
Family Status				.296
Relationship	56.3 (388)	53.3 (112)	57.6 (276)	
No Relationship	43.7 (301)	46.7 (98)	42.4 (203)	
Immigrant Background				.513
Yes	13.9 (96)	15.2 (32)	13.4 (64)	
No	86.1 (593)	84.8 (178)	86.6 (415)	
BMI				<.001
Underweight	4.9 (34)	0.5 (1)	6.9 (33)	
Normal weight	81.8 (563)	81.0 (170)	82.2 (393)	
Overweight	13.2 (91)	18.6 (39)	10.9 (52)	
Kind of University				.301
University	82.7 (570)	84.3 (177)	82.0 (393)	
University of Applied Sciences	12.3 (85)	11.4 (24)	12.7 (61)	
Dual University	0.7 (5)	1.4 (3)	0.4 (2)	
Others	4.2 (29)	2.9 (6)	4.8 (23)	
Field of study				<.001
Politics and Social Sciences	12.5 (86)	9.5 (20)	13.8 (66)	
Education	4.4 (30)	5.7 (12)	3.8 (18)	
Medicine and Health Sciences	53.6 (369)	49.5 (104)	55.3 (265)	
Natural Sciences and Maths	4.8 (33)	6.2 (13)	4.2 (20)	
Law	6.7 (46)	10.0 (21)	5.2 (25)	
Linguistic and Cultural Studies	5.2 (36)	1.9 (4)	6.7 (32)	
Psychology	4.4 (30)	3.3 (7)	4.8 (23)	
Sport Sciences	6.2 (43)	9.0 (19)	5.0 (24)	
Others	2.3 (16)	4.8 (10)	1.3 (6)	
Number of Semesters				.361
1–3	34.9 (234)	31.2 (64)	36.5 (170)	
4–5	18.9 (127)	20.5 (42)	18.2 (85)	
6–9	27.9 (187)	26.8 (55)	28.3 (132)	
10+	18.3 (123)	21.5 (44)	17.0 (79)	
Sport activity per week				<.001
None	8.1 (56)	7.1 (15)	8.6 (41)	
Less than one hour	8.6 (59)	7.6 (16)	9.0 (43)	
1–2 h	17.9 (123)	11.9 (25)	20.5 (98)	
2–4 h	30.3 (209)	23.8 (50)	33.2 (159)	
More than 4 h	35.1 (242)	43.0 (104)	28.8 (138)	
General self-rated general health				.933
Very good/good	86.5 (596)	86.7 (182)	86.4 (414)	
Very poor/poor/fair	13.5 (93)	13.3 (28)	13.6 (65)	
General self-rated mental health				.135
Very good/good	76.3 (526)	80.0 (168)	74.7 (358)	
Very poor/poor/fair	23.7 (163)	20.0 (42)	25.3 (121)	
General self-rated physical health				.299
Very good/good	81.0 (558)	83.3 (175)	80.0 (383)	
Very poor/poor/fair	19.0 (131)	16.7 (35)	20.0 (96)	

**Fig. 1:**
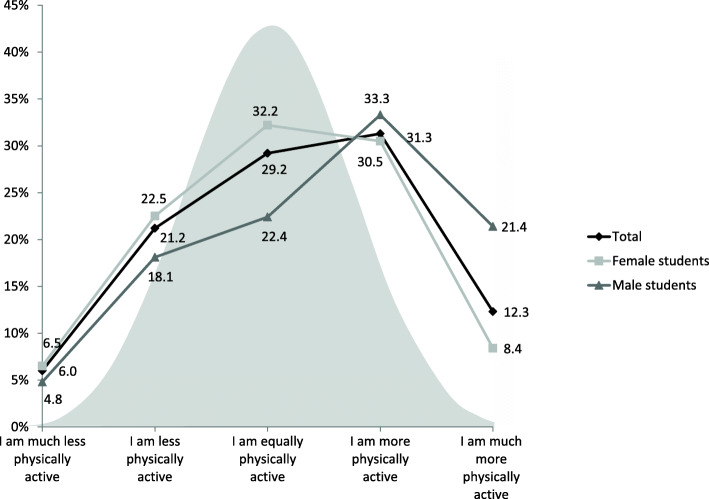
Comparative PA in university students (*n* = 689, NuPhA-Study). PA = physical activity

While 29.2% of the participants indicated being equally physically active compared to fellow students, more students reported being physically more active (more: 31.3%, much more: 12.3%) than less active students (less: 21.2%, much less: 6.0%; Fig. 1). Therefore, we found UCO at the group level, which led to an uneven distribution. This effect was more pronounced in male students than in female students.

The individual assessment with the comparative question seemed appropriate because we found significant associations with paying attention to sufficient PA (*p* < 0.001), self-rated physical performance (*p* < 0.001), PA during the last week (*p* < 0.001), and a normal week (*p* < 0.001), the self-rated regularity of PA (*p* < 0.001), and the amount of sports activity per week (*p* < 0.001, Table 2). In addition, individuals who reported to be (much) less physically active were more likely to have very poor/poor/fair self-rated general (*p* < 0.001), physical (*p* < 0.001), and mental health (*p* < 0.001).

**Table 2:** Association between comparative PA and sport and health related aspects

	I am much less physically active	I am less physically active	I am equally physically active	I am more physically active	I am much more physically active	*p*-value
	% (n)	% (n)	% (n)	% (n)	% (n)	
Paying attention to sufficient PA						<.001
Very much	0.0% (0)	0.7% (1)	6.3% (9)	46.5% (67)	46.5% (67)	
Much	0.9% (2)	5.0% (11)	32.9% (73)	53.6% (119)	7.7% (17)	
Neutral	2.8% (6)	33.5% (72)	49.8% (107)	13.5% (29)	0.5% (1)	
Less	27.8% (25)	57.8% (52)	13.3% (12)	1.1% (1)	0.0% (0)	
Much less	44.4% (8)	55.6% (10)	0.0% (0)	0.0% (0)	0.0% (0)	
Physical performance						<.001
Very good	0.0% (0)	0.8% (1)	9.3% (11)	42.4% (50)	47.5% (56)	
Good	1.3% (4)	11.7% (36)	32.7% (101)	46.0% (142)	8.4% (26)	
Moderate	5.3% (10)	35.8% (67)	44.9% (84)	12.3% (23)	1.6% (3)	
Not really good	30.9% (21)	60.3% (41)	7.4% (5)	1.5% (1)	0.0% (0)	
Not good at all	85.7% (6)	14.3% (1)	0.0% (0)	0.0% (0)	0.0% (0)	
PA during the last week: for at least 60 min on						<.001
0 days	23.2% (26)	54.5% (61)	15.2% (17)	7.1% (8)	0.0% (0)	
1 day	7.7% (8)	37.5% (39)	41.3% (43)	13.5% (14)	0.0% (0)	
2 days	2.2% (3)	17.6% (24)	47.8% (65)	30.9% (42)	1.5% (2)	
3 days	1.5% (2)	11.3% (15)	35.5% (47)	42.9% (57)	9.0% (12)	
4 days	1.1% (1)	5.7% (5)	18.2% (16)	54.5% (48)	20.5% (18)	
5 days	0.0% (0)	0.0% (0)	13.6% (8)	57.6% (34)	28.8% (17)	
6 days	0.0% (0)	2.9% (1)	11.8% (4)	23.5% (8)	61.8% (21)	
7 days	4.3% (1)	4.3% (1)	4.3% (1)	21.7% (5)	65.2% (15)	
PA during a normal week: for at least 60 min on						<.001
0 days	36.8% (25)	48.5% (33)	11.8% (8)	2.9% (2)	0.0% (0)	
1 day	7.7% (8)	49.0% (51)	34.6% (36)	8.7% (9)	0.0% (0)	
2 days	3.4% (5)	27.7% (41)	47.3% (70)	20.9% (31)	0.7% (1)	
3 days	1.5% (2)	8.3% (11)	38.3% (51)	48.1% (64)	3.8% (5)	
4 days	0.8% (1)	2.5% (3)	20.3% (24)	51.7% (61)	24.6% (29)	
5 days	0.0% (0)	10.6% (7)	9.1% (6)	50.0% (33)	30.3% (20)	
6 days	0.0% (0)	0.0% (0)	11.4% (4)	28.6% (10)	60.0% (21)	
7 days	0.0% (0)	0.0% (0)	11.8% (2)	35.3% (6)	52.9% (9)	
The regularity of sport activity with sweating or fast heartbeat						<.001
Often	0.3% (1)	3.0% (10)	22.4% (74)	49.4% (163)	24.8% (82)	
Sometimes	4.3% (11)	31.9% (81)	43.7% (111)	18.9% (48)	1.2% (3)	
Never	30.2% (29)	54.2% (52)	13.5% (13)	2.1% (2)	0.0% (0)	
The amount of sport activity per week						<.001
None	42.9% (24)	50.0% (28)	5.4% (3)	1.8% (1)	0.0% (0)	
1 h	11.9% (7)	62.7% (37)	20.3% (12)	5.1% (3)	0.0% (0)	
1–2 h	5.7% (7)	40.7% (50)	41.5% (51)	11.4% (14)	0.8% (1)	
2–4 h	1.4% (3)	11.0% (23)	48.3% (101)	35.9% (75)	3.3% (7)	
more than 4 h	0.0% (0)	3.3% (8)	14.0% (34)	50.8% (123)	31.8% (77)	
Strenuous PA per week (mean [SD])	0.29 [0.64]	0.92 [1.57]	1.65 [1.19]	2.62 [1.49]	4.11 [2.17]	<.001
Moderate PA per week (mean [SD])	1.20 [2.08]	1.80 [2.27]	2.30 [2.13]	2.45 [2.13]	2.77 [2.53]	<.001
Overall leisure-time activity score (mean [SD])	14.10 [15.39]	23.47 [20.22]	32.67 [17.15]	41.16 [19.28]	55.63 [26.60]	<.001
Health contribution score (mean [SD])	8.61 [12.50]	17.17 [19.08]	26.46 [14.93]	35.81 [16.65]	50.37 [23.05]	<.001
Health contribution score						<.001
Active	1.6% (6)	9.6% (37)	24.5% (104)	42.4% (163)	19.3% (74)	
Moderately active	3.3% (5)	24.8% (38)	39.2% (60)	28.8% (44)	3.9% (6)	
Insufficiently active	21.0% (30)	47.6% (68)	24.5% (35)	6.3% (9)	0.7% (1)	
Self-rated general health						<.001
fair/poor/very poor	18.3% (17)	39.8% (37)	20.4% (19)	17.2% (16)	4.3% (4)	
Very good/good	4.0% (24)	18.3% (109)	30.5% (182)	33.6% (200)	13.6% (81)	
Self-rated mental health						<.001
fair/poor/very poor	11.7% (19)	29.4% (48)	22.1% (36)	28.2% (46)	8.6% (14)	
Very good/good	4.2% (22)	18.6% (98)	31.4% (165)	32.3% (170)	13.5% (71)	
Self-rated physical health						<.001
fair/poor/very poor	19.1% (25)	40.5% (53)	21.4% (28)	16.0% (21)	3.1% (4)	
Very good/good	2.9% (16)	16.7% (93)	31.0% (173)	34.9% (195)	14.5% (81)	

*PA* Physical activity, *SD* Standard deviation

*p*-values are based on chi^2^-statistics (for metric variables: Kruskal-Wallis-H-test)

Data drawn from the cross-sectional Nutrition and Physical Activity in Adolescence Study (NuPhA)

Overall leisure-time activity score, health contribution score, and categories based on the health contribution score are based on the Godin-Shepard leisure-time PA questionnaire (Godin 2011 [15])

**Fig. 2:**
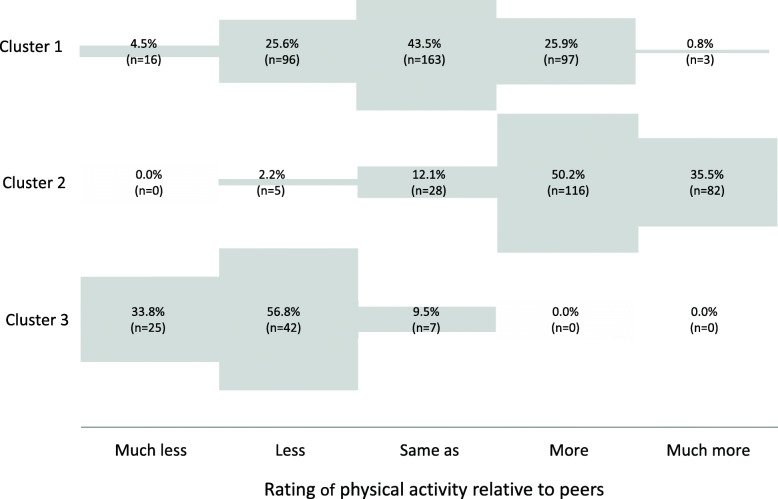
Results of the cluster analysis by comparative PA (*n* = 689, NuPhA-Study). PA = physical activity

The cluster analysis based on the above-mentioned PA variables revealed three clusters (Fig. 2). Cluster 1 reflects a normal distribution regarding the comparative measure of PA. Cluster 2 includes predominantly individuals who were (much) more physically active than same-aged fellow students. Cluster 3 describes students who reported to be (much) less physically active.

These three clusters seem to be plausible, for instance when compared with the distribution of the regularity of sports activity resulting in sweating or fast heartbeat. While all individuals of cluster 3 (100%) indicated that they never or hardly reached this level of sports activity, the majority of cluster 2 (94.4%) reported that they often perform sports activities with sweating or fast heartbeat. Cluster 2 included a higher number of individuals than Cluster 3 (*n* = 231 vs. *n* = 74). This explains the UCO at the group level, with more individuals indicating to be more physically active than individuals indicating to be less physically active (Fig. 1). The majority in cluster 1 (64.3%) sometimes performed sports activities with sweating or fast heartbeats.

## Discussion

In summary, we observed UCO at the group level, with more students indicating to be more physically active compared to average than students indicating to be less physically active, and this led to a non-normal distribution. However, our cluster analysis and associations with other variables on PA, sports activity, and subjective health demonstrated that this shift to more PA seems reasonable, because our sample is a very physically active group with only 8.1% reporting to be physically inactive. The cluster analysis revealed three clusters: The numeric biggest cluster (Cluster 1), including those with a medium level of PA, showed the expected normal distribution. In addition, we identified a very active cluster (Cluster 2), which included a higher number of students than the cluster that included the physically inactive students (Cluster 3). Therefore, the non-normal distribution seems plausible, given that students compared themselves to a student with medium PA. This leads to the conclusion that the suggested comparative measurement by Sternfeld et al. [2] seems to reflect the actual self-reported PA of students.

When answering comparison questions, it is often difficult for respondents to identify the comparison standard they need a frame of reference. Lechner et al. [22] wrote that people with misperceptions more often use downward comparison and therewith use a comparison target that behaves less healthy (here: being less physically active). One reason for this could be self-enhancement [22, 23]. Nonetheless, a correct assessment of one’s own PA and the awareness of personal risk behavior are essential for behavioral change [22, 24]. If individuals (incorrectly) believe that their behavior is already healthy and adequate, they are less convinced by intervention measures [1, 22, 25, 26].

Although we found UCO at the group level, that is, a non-normal distribution of the answers to the PA comparison question, our cluster analysis revealed that inactive students were able to correctly assess their (low) PA in the comparative question. Following models of behavioral change [27], such a correct self-assessment, should be the first step towards changing intentions and behavior regarding PA, to enhance PA.

In our sample, only a minority of the participants were physically inactive, which suggests the need to investigate UCO in less active and clinical study populations in future studies. When using a comparative question on PA in future research, it is highly recommended to include an objective measurement of PA. This will allow testing criterion validity of the comparative measure. Such studies will allow us to conclude whether a single comparison question is a useful measurement for PA as a covariate in large surveys. Our study provides important groundwork since we found associations with other self-reported PA and health measures.

### Strengths and limitations

To our knowledge, this is the first time that a comparative question on PA was used in a large quantitative sample of university students. We were able to assess a variety of aspects regarding PA, sports, and health to investigate associations with the comparative assessment. Nonetheless, our results should be interpreted against the backdrop of potential weaknesses.

First, due to the cross-sectional design, our study does not allow us to draw any conclusions on causality or causal directions of the observed relations. Furthermore, because all variables are self-reported, social desirability and recall bias may occur. In addition, data was already collected between 2014 and 2015. However, since our aim was not to provide current prevalence on PA in students, but to investigate a psychological phenomenon, this aspect might be negligible. In our manuscript, we compared different self-reported measures of PA with each other. The gold standard would be to have an objective measure, for instance, accelerometry, to draw conclusions regarding criterion validity. To have at least an established and validated measure, we included the widely used Godin-Shephard leisure-time PA questionnaire, which enabled us at least to analyze concurrent validity.

Moreover, since our aim was to collect data of students from all over Germany, we did not follow a university-specific name list. Therefore, we are not able to calculate a response rate and thus, a potential participation bias cannot be completely excluded. Among our participants, we have a large group of students studying medicine, health science or sport science. We cannot exclude that these students are more likely to be physically active due to their knowledge about the potential health consequences of physical inactivity. Altogether, these biases may influence the generalizability of our results. However, our primary focus was not on presenting nationwide representative data, but rather to contribute to the validation of the comparative measurement of PA in a specific target group of university students.

## Conclusion

Overall, the comparative single question used by Sternfeld et al. [2] seems to be a valid measure in university students, based on our comparison with other self-reported measures. Supported by our results, the students in this sample were able to rate their individual PA realistically, but UCO was found at the group level. However, this UCO might attributed to the overall high physical activity of the students in our sample.

In addition to our study, it would be interesting to use this single comparison question in a larger, more general population and in clinical samples to determine whether people are able to rate themselves realistically. Based on these results, possible intervention programs and tailored health promotion strategies can be generated.

## Supplementary Information

**Additional file 1.**

## Data Availability

The dataset analyzed during the current study is not publicly available but is available from the corresponding author on reasonable request.

## Abbreviations

UCO: Unrealistic comparative optimism
PA: Physical activity

## References

[1] van Sluijs E, Griffin S, van Poppel M (2007). A cross-sectional study of awareness of physical activity: associations with personal, behavioral and psychosocial factors. Int J Behav Nutr Phys Act.

[2] Sternfeld B, Cauley J, Harlow S, Liu G, Lee M (2000). Assessment of physical activity with a single global question in a large, multiethnic sample of midlife women. Am J Epidemiol.

[3] Caspersen CJ, Powell KE, Christenson GM (1985). Physical activity, exercise, and physical fitness: definitions and distinctions for health-related research. Public Health Rep.

[4] Milton K, Clemes S, Bull F (2013). Can a single question provide an accurate measure of physical activity?. Br J Sports Med.

[5] Shepperd JA, Klein WM, Waters EA, Weinstein ND (2013). Taking stock of unrealistic optimism. Perspect Psychol Sci.

[6] Weinstein ND (1980). Unrealistic optimism about future life events. J Pers Soc Psychol.

[7] Burger JM, Burns L (1988). The illusion of unique invulnerability and the use of effective contraception. Personal Soc Psychol Bull.

[8] Weinstein ND (1982). Unrealistic optimism about susceptibility to health problems. J Behav Med.

[9] Shepperd JA, Waters E, Weinstein ND, Klein WM (2015). A primer on unrealistic optimism. Curr Dir Psychol Sci.

[10] Krug S, Jordan S, Mensink GB, Muters S, Finger J, Lampert T (2013). Physical activity: results of the German health interview and examination survey for adults (DEGS1). Bundesgesundheitsblatt Gesundheitsforschung Gesundheitsschutz.

[11] Tittlbach S, Sygusch R, Brehm W, Seidel I, Bös K (2010). Physical education: Chance for health for inactive children and adolescents?. Sportwissenschaft..

[12] Prochaska JJ, Sallis JF, Long B (2001). A physical activity screening measure for use with adolescents in primary care. Arch Pediatr Adolesc Med.

[13] Godin G, Shephard RJ (1985). A simple method to assess exercise behavior in the community. Can J Appl Sport Sci.

[14] Godin G, Shephard RJ (1997). Godin Leisure-Time Exercise Questionnaire. Med Sci Sport Exerc.

[15] Godin G (2011). The Godin-Shephard leisure-time physical activity questionnaire. HFJC..

[16] Babenko O, Mosewich A, Abraham J, Lai H (2018). Contributions of psychological needs, self-compassion, leisure-time exercise, and achievement goals to academic engagement and exhaustion in Canadian medical students. J Educ Eval Health Prof.

[17] Liu KT, Kueh YC, Arifin WN, Kim Y, Kuan G (2018). Application of Transtheoretical Model on Behavioral Changes, and Amount of Physical Activity Among University's Students. Front Psychol.

[18] Amireault S, Godin G (2015). The Godin-Shephard leisure-time physical activity questionnaire: validity evidence supporting its use for classifying healthy adults into active and insufficiently active categories. Percept Mot Skills.

[19] Jacobs DR, Ainsworth BE, Hartman TJ, Leon AS (1993). A simultaneous evaluation of 10 commonly used physical activity questionnaires. Med Sci Sports Exerc.

[20] Miller DJ, Freedson PS, Kline GM (1994). Comparison of activity levels using the Caltrac(R) accelerometer and 5 questionnaires. Med Sci Sport Exer.

[21] Aldenderfer MS, Blashfiedl RK (1984). Cluster analysis.

[22] Lechner L, Bolman C, Van Dijke M (2006). Factors related to misperception of physical activity in the Netherlands and implications for health promotion programmes. Health Prom Internat.

[23] Oenema A, Brug J (2003). Exploring the occurrence and nature of comparison of one's own perceived dietary fat intake to that of self-selected others. Appetite..

[24] Ronda G, Van Assema P, Brug J (2001). Stages of change, psychological factors and awareness of physical activity levels in the Netherlands. Health Promot Int.

[25] White MS, Addison CC, Jenkins BW, Bland V, Clark A, LaVigne DA (2017). Optimistic Bias, risk factors, and development of high blood pressure and obesity among African American adolescents in Mississippi (USA). Int J Environ Res Public Health.

[26] Arnett JJ (2000). Optimistic bias in adolescent and adult smokers and nonsmokers. Addict Behav.

[27] Fishbein M, Ajzen I (1975). Belief, attitude, intention, and behavior : an introduction to theory and research.

