# Supplementary material for: Comparative physical activity as a global question to assess physical activity among university students
Source: BMC Sports Sci Med Rehabil. 2021 Mar 2;13:19. doi: 10.1186/s13102-021-00247-7 (PMC7923472; doi:10.1186/s13102-021-00247-7)
Supplement: Supplementary file 1 — Additional file 1. [file 13102_2021_247_MOESM1_ESM.docx]

**Supplement 1: Abstract of the questionnaire (questions on physical activity)**

**Compared to your same-aged fellow students are you …**

| _1_ | _2_ | _3_ | _4_ | _5_ |  |
| --- | --- | --- | --- | --- | --- |
| much less | less | same as | more | much more | **… physically active?** |

*(used in German; original source: Sternfeld et al. 2000 (1))*

**How do you rate your physical performance?**

| ☐1 | ☐2 | ☐3 | ☐4 | ☐5 |
| --- | --- | --- | --- | --- |
| **Very good** | **Good** | **Moderate** | **Not really good** | **Not good at all** |

*(used in German; original source: German Health Interview and Examination Survey for Children and Adolescents (KiGGS))*

**Overall, how much do you pay attention to getting enough physical activity?**

☐1 very much

☐2 much

☐3 neutral

☐4 less

☐5 much less

*(used in German; original source: German Health Interview and Examination Survey for Adults (DEGS1))*

**Physical activity is any activity that increases your heart rate and makes you get out of breath some of the time.**

**Physical activity can be done in sports, playing with friends, or walking to school.**

**Some examples of physical activity are running, brisk walking, rollerblading, biking, skateboarding, dancing, swimming, soccer, basketball, football, & surfing**

**Add up all the time you spend in physical activity each day (don’t include your physical education or gym class).**

**Over the past 7d, on how many days were you physically active for a total of at least 60 min per day?**

| ☐1 | ☐2 | ☐3 | ☐4 | ☐5 | ☐6 | ☐7 | ☐8 |
| --- | --- | --- | --- | --- | --- | --- | --- |
| **0 days** | **1** | **2** | **3** | **4** | **5** | **6** | **7 days** |

**Over a typical or usual week, on how many days are you physically active for a total of at least 60 min per day?**

| ☐1 | ☐2 | ☐3 | ☐4 | ☐5 | ☐6 | ☐7 | ☐8 |
| --- | --- | --- | --- | --- | --- | --- | --- |
| **0 days** | **1** | **2** | **3** | **4** | **5** | **6** | **7 days** |

*(used in German; original source: Prochaska et al. 2001 (2))*

**All in all, how many hours per week do you perform sports?**

☐1 I do not perform sports

☐2 Up to one hour

☐3 Between one and two hours

☐4 Between two and four hours

☐5 More than four hours

*(used in German; original source: German Health Interview and Examination Survey for Adults (DEGS1))*

**During a typical 7-Day period (a week), in your leisure time, how often do you engage in any regular activity long enough to work up a sweat (heart beats rapidly)?**

| ☐1 | ☐2 | ☐3 |
| --- | --- | --- |
| **Often** | **Sometimes** | **Never/rarely** |

*(used in German; original source:Godin and Shephard 1985 (3))*

**During a typical 7-Day period (a week), how many times on the average do you do the following kinds of exercise for more than 15 minutes during your free time.**

|  | Times per week |
| --- | --- |
| STRENUOUS EXERCISE (HEART BEATS RAPIDLY)  (e.g., running, jogging, hockey, football, soccer, squash, basketball, cross country skiing, judo, roller skating, vigorous swimming, vigorous long distance bicycling) | _____ |
| MODERATE EXERCISE (NOT EXHAUSTING)  (e.g., fast walking, baseball, tennis, easy bicycling,volleyball, badminton, easy swimming, alpine skiing, popular and folk dancing) | _____ |
| MILD EXERCISE (MINIMAL EFFORT)  (e.g., yoga, archery, fishing from river bank, bowling, horseshoes, golf, snow-mobiling, easy walking) | _____ |

*(used in German; original source:Godin 2011 (4))*

1. Sternfeld B, Cauley J, Harlow S, Liu G, Lee M. Assessment of physical activity with a single global question in a large, multiethnic sample of midlife women. Am J Epidemiol. 2000;152:678-87.

2. Prochaska JJ, Sallis JF, Long B. A physical activity screening measure for use with adolescents in primary care. Arch Pediatr Adolesc Med. 2001;155:554-9.

3. Godin G, Shephard RJ. A simple method to assess exercise behavior in the community. Can J Appl Sport Sci. 1985;10:141-6.

4. Godin G. The Godin-Shephard Leisure-Time Physical Activity Questionnaire. HFJC. 2011;4:18-22.
